# Neonatal Purpura Fulminans: An Unusual Cause of Leukocoria, Retinal Detachment, and Vitreous Hemorrhage in Neonates

**DOI:** 10.7759/cureus.102044

**Published:** 2026-01-21

**Authors:** Krishna K Kadari, Tanushree Sahoo, Bruttendu Moharana, Pankaj Kumar Mohanty, Tapas Kumar Som, Jagdish P Sahoo, Usha Devi

**Affiliations:** 1 Pediatrics, All India Institute of Medical Sciences, Bhubaneswar, Bhubaneswar, IND; 2 Neonatology, All India Institute of Medical Sciences, Bhubaneswar, Bhubaneswar, IND; 3 Ophthalmology, All India Institute of Medical Sciences, Bhubaneswar, Bhubaneswar, IND

**Keywords:** coagulopathy, congenital disorder, purpura, retinal detachment (rd), skin lesion

## Abstract

Neonatal purpura fulminans is a rare but life-threatening thrombotic disorder caused by congenital deficiencies of protein C. It presents with hemorrhagic necrosis of the skin due to the underlying dysfunction of coagulation. Ocular findings include leukocoria, chemosis, periorbital edema, posterior synechia, vitreous hemorrhage, retinal detachment, and retinal dysplasia. Sometimes, leukocoria may occur before any other manifestations. Here, we describe a case of neonatal purpura fulminans characterized by multiple purpuric skin lesions, leukocoria, vitreous hemorrhage, and total retinal detachment.

## Introduction

Neonatal purpura fulminans is a rare progressive, thrombotic disorder arising from congenital deficiencies in protein C. It is an autosomal recessive disorder following a homozygous or compound heterozygous mutation in the PROC gene, with an incidence of one in four million [1]. The clinical manifestation of neonatal purpura fulminans is manifested by the rapid development of hemorrhagic necrosis in the skin due to underlying coagulation dysfunction. Additionally, ocular complications are prevalent and encompass symptoms such as leukocoria, chemosis, periorbital edema, posterior synechia, vitreous hemorrhage, retinal detachment, and retinal dysplasia [1,2].

In this context, we present an interesting case of neonatal purpura fulminans with severe visual involvement along with skin lesions. This description underscores the severity and multifaceted nature of the condition, necessitating prompt recognition and intervention for effective management.

## Case presentation

The infant was a single, term (39 weeks), appropriate-for-gestational-age (2.5 kg) male newborn with an asymptomatic antenatal period, born to parents with third-degree consanguinity. Except for the history of intrauterine death in the previous pregnancy at eight months of gestation, the rest of the antenatal period was uneventful. The peripartum period was asymptomatic, and the infant was nursed with the mother in the postnatal ward. The parents noticed multiple purpuric patches over the newborn's body involving limbs, trunk, and buttocks on day 3 of life. The infant was evaluated for the same elsewhere. Complete blood count and coagulogram suggested features of disseminated coagulopathy with consumptive thrombocytopenia. Blood culture as part of the sepsis workup suggested *Staphylococcus aureus* septicemia, for which he received appropriate antibiotics based on culture sensitivity. For coagulopathy, he received four random donor platelet infusions and fresh frozen plasma (FFP) during his hospital stay. However, the skin lesions persisted. New skin lesions also appeared. He was admitted to our neonatal intensive care unit (NICU) on day 14 of life for further management.

**Figure 1:**
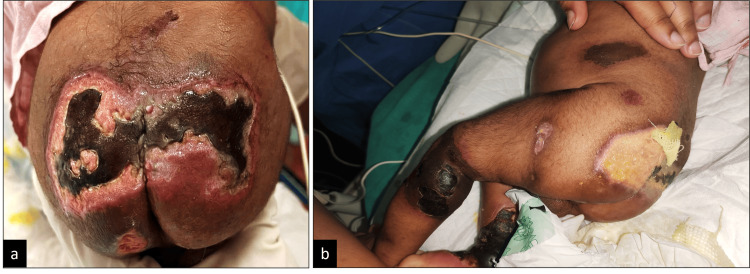
Gangrenous skin lesions in various stages of healing on the buttock (a) and lower limbs and abdomen (b).

**Figure 2:**
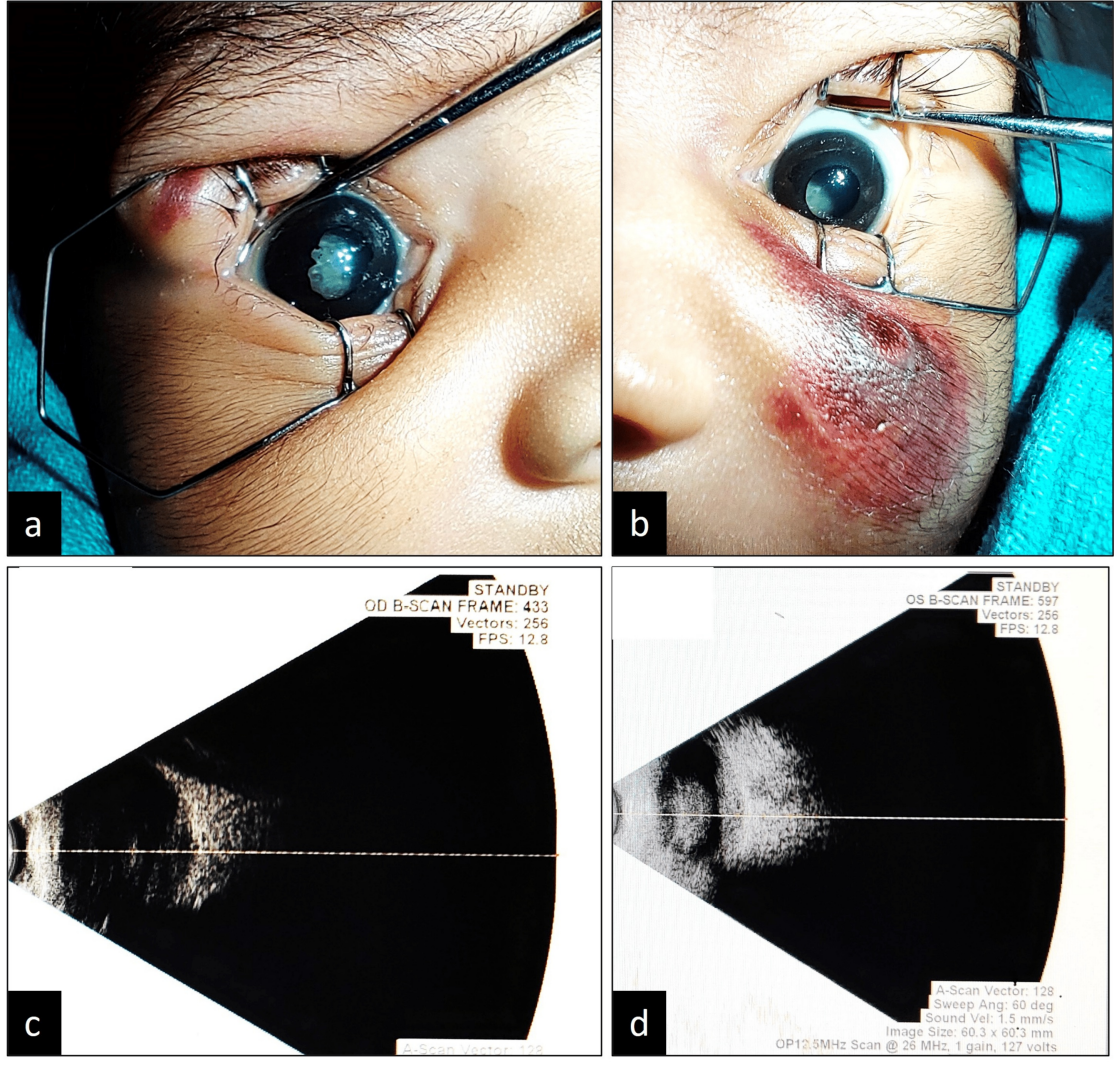
Bilateral white pupillary reflexes and pupillary synechiae (a, b). Ultrasound B-scan demonstrates moderate-to-high-intensity echoes suggestive of vitreous hemorrhage and retinal detachment (c, d).

On admission, the baby was hemodynamically stable. There were multiple purpuric lesions of various stages of progression all over the body (Figures 1a, 1b). On ocular examination, bilateral white reflexes were noticed. The patient was evaluated by a vitreo-retinal specialist. Pupillary synechiae were noticed in the right eye (RE) (Figure 2a). A bilateral, retro-lenticular, white, membranous structure with blood vessels was noticed, suggesting total retinal detachment (Figures 2a, 2b).

Investigations

The neonate was investigated for common causes of purpura, including neonatal sepsis, coagulation disorders, liver dysfunction (acute or chronic), vascular disorders, platelet disorders, and inherited thrombophilia due to deficiencies of protein C, protein S, fibrinogen, and antithrombin III. The sepsis workup was negative. The coagulation profile and liver function tests were normal. Abdominal ultrasound findings were unremarkable. To exclude inherited thrombophilia secondary to deficiencies of protein C/S, fibrinogen, and antithrombin III, appropriate investigations were sent, which revealed severe protein C deficiency (factor C level was 0%), thus confirming the provisional diagnosis of congenital protein C deficiency. Clinical exome sequencing in the index case identified a homozygous mutation in exon 5 of the PROC gene on chromosome 2 (c.315C>A, p.Cys105Ter), confirming thrombophilia type 3 due to protein C deficiency, culminating in neonatal purpura fulminans. Ophthalmic ultrasound revealed dot-like echogenicity in the vitreous cavity bilaterally, suggesting vitreous hemorrhage. The presence of a high echogenic membranous structure suggested a total retinal detachment (Figure 2c).

Treatment

As recombinant protein C replacement therapy was not available at our hospital, supportive treatment with daily FFP transfusions was initiated and later escalated to two to three times daily during admission. The infant also received subcutaneous low-molecular-weight heparin; however, new lesions continued to appear.

Outcomes and follow-up

The parents were informed about the nature of the disease, the need for long-term protein C, and anticoagulant prophylaxis till definitive therapy in the form of a liver transplant. Poor visual prognosis was also explained to them because of bilateral complete retinal detachment and vitreous hemorrhage. However, the parents subsequently decided against the continuation of ongoing care. Hence, the patient was discharged on request on day 11 of his hospital stay. His parents were advised to continue anticoagulant therapy. Parents were also counseled regarding the need for detailed antenatal genetic workup for future pregnancies. On telephonic follow-up after 15 days, the parents informed us that the baby had passed away at home.

## Discussion

Protein C is a vitamin K-dependent anticoagulant that, in its activated form, prevents the formation of blood clots by down-regulating the coagulation cascade and blocking the spread of thrombosis [3]. Though neonatal purpura fulminans is usually a severe manifestation of congenital homozygous protein C deficiency, many times it follows as secondary deficiencies of protein C, protein S, and antithrombin III following Gram-negative fulminant sepsis due to consumptive coagulopathy. Congenital protein C deficiency typically presents within a few hours of birth with cutaneous infarcts and features of diffuse intravascular coagulation (DIC) [1].

Reports on ocular involvement in protein C deficiency are not very common; rarely, it can cause leukocoria, vitreous hemorrhage, and total retinal detachment at birth. Various ophthalmic manifestations include non-reactive pupils, periorbital edema, shallow anterior chamber, posterior synechia, microphthalmos, vitreous hemorrhage, retinal and subretinal hemorrhage, retinal arterial and venous occlusion, and retinal dysplasia [2,4,5]. It may be unilateral or bilateral. Sometimes, leukocoria may be the presenting sign of homozygous protein C deficiency before the appearance of purpura fulminans. Thrombosis of fetal hyaloid vessels can cause arrested ocular development, leading to structural manifestations like microphthalmos, persistent primary hyperplastic vitreous, incomplete retinal vascularization, vascular leakage, and retinal detachment [2]. Retinal detachment, vitreous hemorrhage, and subretinal hemorrhage can occur in utero. Such complications can present with leukocoria at birth, with the development of skin lesions later.

On diagnosis of homozygous protein C deficiency, treatment should start promptly by infusing FFP or protein C concentrate. Appropriate antithrombotic drugs like low-molecular-weight heparin should be administered. The treatment during the acute phase should continue until all lesions resolve. Anticoagulation and protein C concentrate should continue as maintenance therapy. As protein C is produced in the liver, a non-related healthy liver transplant may provide a definitive management option by subsequently producing an adequate amount of protein C. Most ocular manifestations may not be amenable to any definitive management. Incomplete retinal vascularization may be treated with laser photocoagulation, which may help preserve useful vision in the patient [2].

Leukocoria in newborns can occur due to cataracts, persistent hyperplastic primary vitreous, retinal detachments, and retinoblastoma. The clinical signs of homozygous protein C deficiency manifest from two hours to two weeks after birth. Having a high index of suspicion towards this unusual cause of leukocoria at birth, the condition can be detected early, and prompt treatment can be started.

## Conclusions

This case report highlights that leukocoria at birth may indicate an underlying systemic thrombophilia, such as congenital protein C deficiency. Leukocoria can sometimes be the only presenting complaint. A high index of suspicion followed by early diagnosis and plasma replacement is critical to survival. Genetic counseling and screening are essential for families with a history of neonatal purpura fulminans. However, the inherent limitation of a case report is acknowledged, and a further high-volume observational study is warranted to establish such a conclusion firmly.
